# Neuroprotective Roles of the Biliverdin Reductase-A/Bilirubin Axis in the Brain

**DOI:** 10.3390/biom14020155

**Published:** 2024-01-28

**Authors:** Bindu D. Paul, Andrew A. Pieper

**Affiliations:** 1 Department of Pharmacology and Molecular Sciences, Johns Hopkins University School of Medicine, Baltimore, MD 21205, USA; 2 The Solomon H. Snyder Department of Neuroscience, Johns Hopkins University School of Medicine, Baltimore, MD 21205, USA; 3 Department of Psychiatry and Behavioral Sciences, Johns Hopkins University School of Medicine, Baltimore, MD 21205, USA; 4 Lieber Institute for Brain Development, Baltimore, MD 21205, USA; 5 Department of Psychiatry, Case Western Reserve University, Cleveland, OH 44106, USA; 6 Brain Health Medicines Center, Harrington Discovery Institute, University Hospitals Cleveland Medical Center, Cleveland, OH 44106, USA; 7 Geriatric Psychiatry, GRECC, Louis Stokes Cleveland VA Medical Center, Cleveland, OH 44106, USA; 8 Institute for Transformative Molecular Medicine, School of Medicine, Case Western Reserve University, Cleveland, OH 44106, USA; 9 Department of Pathology, School of Medicine, Case Western Reserve University, Cleveland, OH 44106, USA; 10 Department of Neurosciences, School of Medicine, Case Western Reserve University, Cleveland, OH 44106, USA

**Keywords:** bilirubin, biliverdin reductase, superoxide, lipophilic antioxidant, cognition

## Abstract

Biliverdin reductase-A (BVRA) is a multi-functional enzyme with a multitude of important roles in physiologic redox homeostasis. Classically, BVRA is well known for converting the heme metabolite biliverdin to bilirubin, which is a potent antioxidant in both the periphery and the brain. However, BVRA additionally participates in many neuroprotective signaling cascades in the brain that preserve cognition. Here, we review the neuroprotective roles of BVRA and bilirubin in the brain, which together constitute a BVRA/bilirubin axis that influences healthy aging and cognitive function.

## 1. Introduction

Biliverdin reductase (BVR) was discovered in 1936 when Lemberg and Wyndham observed that crude liver extracts convert green biliverdin, the product of heme metabolism, into a yellow colored substance [1]. They termed this “biliverdin reductase activity.” Much later, in 1965, the enzyme responsible for this activity was partially purified from guinea pig liver [2]. Subsequently, in 1981 and 1993, Maines and colleagues purified BVR to homogeneity from rat and human liver, respectively, and fully characterized its enzymatic activity [3,4].

**Figure 1:**
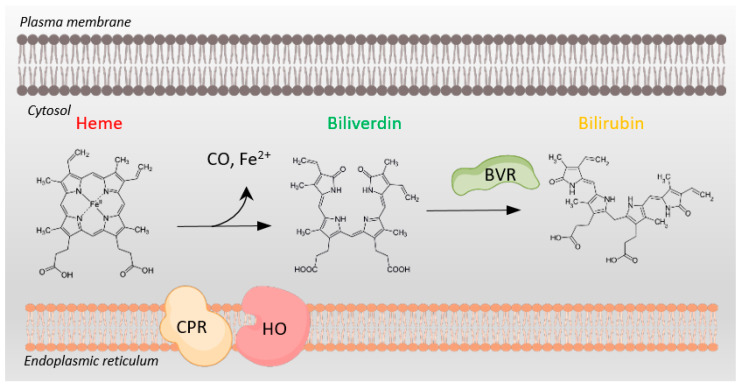
Heme metabolism requires NADPH oxidation. Conversion of cytosolic heme to biliverdin by heme oxygenase (HO) and cytochrome P450 reductase (CPR), both tethered to the endoplasmic reticulum membrane, involves NADPH oxidation to NADP+. Subsequent conversion of biliverdin to bilirubin by biliverdin reductase (BVR) also depends on NADPH oxidation to NADP+. BVR plays multiple additional roles in cellular physiology as well.

Today, BVR is well known as a key enzyme in heme catabolism. Specifically, after heme oxygenases cleave heme to biliverdin, with a concomitant release of iron and carbon monoxide, BVR rapidly reduces biliverdin to bilirubin in a reaction coupled to the oxidation of the pyridine nucleotide cofactors NADH and NADPH (Figure 1). An intriguing feature of BVR is that it utilizes these dual cofactors at two different pH optima: NADH at pH 6.7 and NADPH at pH 8.7 [3,4]. This dual pH usage allows BVR to function in different cellular compartments in varying physiological states of altered pH [5]. BVR exists as two functional isoforms, BVRA and BVRB, with BVRB expression emerging at 14–15 weeks of age and BVRA expression not until closer to 20 weeks [6]. BVRB is also predominantly expressed in fetal tissues, while BVRA is the predominant form present in adults and the major enzyme for bilirubin production across our lifespan [7,8]. Importantly, bilirubin is cytoprotective, with in vitro and in vivo antioxidant activity [8,9,10,11,12,13,14,15], and BVRA also possesses multiple activities that play additional roles in cellular protection.

Although bilirubin is one of the most frequently measured blood metabolites in a clinical setting, its physiological functions are still not completely understood. To date, most studies on bilirubin in the brain have focused on peripheral body systems and the consequences of accumulating toxic levels of bilirubin in the brain, while the role of bilirubin in protecting the brain at normal physiologic levels has been largely ignored. Here, we review the neuroprotective roles of BVRA and bilirubin, with specific focus on cognitive function in neurodegenerative conditions.

## 2. Bilirubin in the Brain: The Good, the Bad, and the Ugly

The fact that bilirubin production has been retained by a diverse set of organisms despite its high energetic cost suggests important physiologic functions. The production of bilirubin is inducible and tightly controlled, and it exhibits circadian rhythms and is spatially and temporally controlled [16,17]. Upon the breakdown of heme, bilirubin initially exists in its indirect (unconjugated) albumin-bound form, rendering it lipid-soluble and water-insoluble. For bilirubin to be excreted into bile for elimination from the body, it must be converted to its direct (conjugated) water-soluble and less lipid-soluble form. This occurs via conjugation in the liver to glucuronic acid by uridine diphosphate glucuronosyltransferase (UGT1A1) [18]. Also in the liver, microsomal cytochrome P450 2A5 (CYP2A5) acts as an inducible bilirubin oxidase to convert bilirubin to biliverdin [19]. Notably, unconjugated bilirubin is yellow, while conjugated bilirubin is not. Clinically, unconjugated bilirubin is elevated in hemolytic anemia, as well as various inherited disorders such as Gilbert syndrome and Crigler–Najjar syndromes type I and II. Elevated conjugated bilirubin, by contrast, is found in disorders such as obstructive jaundice and biliary atresia. Due to differences in lipid solubility, unconjugated bilirubin passes across the blood–brain barrier (BBB), while conjugated bilirubin cannot.

Bilirubin predominantly circulates in the blood in its unconjugated form, with only a minor (0.01%) conjugated fraction [20]. Historically, increased levels of bilirubin have been traditionally discussed in terms of toxicity. However, normal and mildly elevated levels of bilirubin also serve a less well-recognized normal protective role. For example, mild bilirubin elevation in Gilbert syndrome (GS), which is caused by variants in the gene encoding UGT1A1, is correlated with a reduced incidence of kidney and cardiovascular disease, diabetes, metabolic syndrome, and some forms of cancer [21,22,23]. Additionally, some patients with GS also display improved antioxidant status and decreased levels of pro-inflammatory and pro-oxidant markers, including interleukin 6 (IL-6), IL-1β, apolipoprotein B (Apo B), and C-reactive protein (CRP), compared to study subjects with normal bilirubin levels. The protective effect of bilirubin is especially prominent in the fourth to sixth decade of life [24]. By contrast, the much higher plasma and tissue bilirubin levels in Crigler–Najjar syndrome (CN syndrome), which is characterized by myriad genetic aberrations that result in a near total lack of UGT1A1 activity, are toxic [25,26]. Here, the resulting accumulation of unconjugated bilirubin leads to encephalopathy (kernicterus) and bilirubin-induced neurologic damage (BIND) [27,28]. The consequences can be extreme, such as with CN syndrome type I, which appears shortly after birth, with serum bilirubin approaching 20 to 50 mg/dl, and causes encephalopathy and death. Complications caused by neonatal hyperbilirubinemia, collectively termed kernicterus spectrum disorder (KSD), encompass bilirubin neurotoxicity, acute bilirubin encephalopathy (ABE), chronic bilirubin encephalopathy (CBE), kernicterus, and BIND [27]. The symptoms of KSD generally include impaired motor control and abnormal movements, auditory processing disturbance that may be accompanied by hearing loss, impaired oculomotor function with paralysis of the upward vertical gaze (setting sun sign), and enamel dysplasia of deciduous (baby) teeth [28]. Hyperbilirubinemia can be controlled by aggressive phototherapy to isomerize unconjugated bilirubin to render it excretable from the body, bypassing the need for hepatic conjugation, or by exchange transfusions in neonates [29]. It has been speculated that an isomerization of bilirubin may reduce its lipophilicity and decrease its ability to cross the blood–brain barrier (BBB), but this has not been established experimentally [30,31,32]. In an informative review by Hansen, the clinical implications of photoisomerization therapy have been extensively discussed [31].

**Figure 2:**
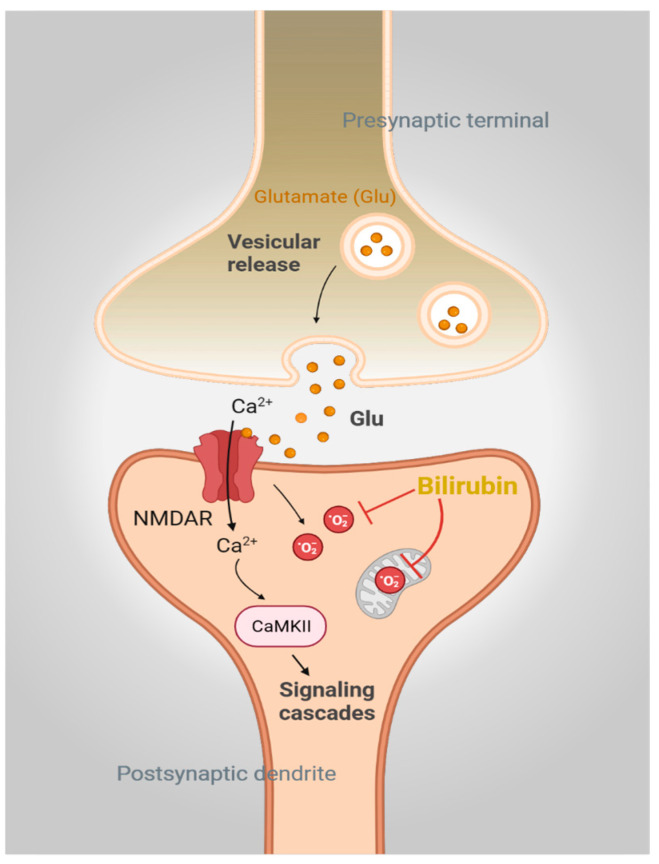
Bilirubin is a physiologic antioxidant. Activation of *N*-methyl-D-aspartate receptor (NMDAR) signaling by glutamate mediates calcium (Ca^2+^) influx and activates downstream signaling pathways involving CaMKII. NMDAR engagement also generates superoxide (O_2_^·−^), which, if not controlled, will lead to neurotoxicity. Bilirubin directly scavenges O_2_^·−^ and prevents NMDAR-mediated neurotoxicity in neurons.

Bilirubin may also exert beneficial effects at physiological concentrations. Studies from our group have shown that the brain actively synthesizes bilirubin with potent neuroprotective effects [8]. For example, bilirubin is a highly effective scavenger of superoxide radicals (O_2_^·−^), both in vitro and in vivo. This scavenging activity plays a central role in *N*-methyl D-aspartate (NMDA)-mediated neurotransmission by preventing the accumulation of reactive oxygen species (ROS) [8] (Figure 2). Although normal NMDAR signaling is in part dependent on O_2_^·−^, excessive NMDAR activity with an excessive accumulation of O_2_^·−^ mediates uncontrolled lipid peroxidation and neuronal death [29]. Indeed, pathologic NMDA receptor activation and lipid peroxidation occur in several neurodegenerative diseases [30,31,32,33]. Notably, cells and neurons derived from mice lacking BVRA (*Blvra*^−/−^) are hypersensitive to oxidative stress induced by O_2_^·−^. *Blvra*^−/−^ cells are more sensitive to several superoxide cyclers, as compared to other oxidants and electrophiles [8]. Thus, mouse embryonic fibroblasts (MEFs) derived from *Blvra*^−/−^ mice are highly prone to oxidative death and are especially sensitive to the O_2_^·−^ donors pyrogallol, paraquat, and menadione. These MEFs were relatively mildly sensitive to the oxidant hydrogen peroxide (H_2_O_2_) and the electrophile 4-hydroxynonenal (4-HNE). These MEFs were also found to be less efficient in clearing ROS as compared to wild-type WT MEFs when exposed to menadione, as revealed by the fluorescent redox probe dihydroethidium (DHE) [8]. Additionally, NMDA treatment and O_2_^·−^ production cause exacerbated excitotoxicity and brain lesions in *Blvra*^−/−^ mice [8]. These effects were also observed in ex vivo brain slices [34].

Although superoxide dismutases (SODs) scavenge O_2_^·−^, these enzymes are specifically localized to soluble compartments in the cell [35]. However, O_2_^·−^ is often produced in aprotic and membrane-rich regions of cells, where it can be readily acted upon by lipophilic bilirubin. As O_2_^·−^ has a negative charge, its passive diffusion out of the cell is prevented, and thus, it accumulates in the mitochondrial matrix or intramembranous spaces. Using mitoUnaG, a mitochondrially targeted UnaG that binds bilirubin and fluoresces, we have shown that bilirubin is present in mitochondria, a site responsible for the generation of O_2_^·−^ when electrons escape from the electron transport chain complexes I-III [8,36]. Notably, mitochondrial O_2_^·−^ play central roles in neuronal death in several conditions, such as ischemic stroke [37]. MEFs derived from *Blvra*^−/−^ mice are highly sensitive to rotenone, an inhibitor of complex I. *Blvra*^−/−^ MEFs exhibited higher levels of O_2_^·−^ as compared to their wild-type controls despite the comparable expression of SODs and component proteins of the electron transport chain [8]. Furthermore, incubating these MEFs with bilirubin substantially decreased O_2_^·−^ levels. Along similar lines, cerebella of *Blvra*^−/−^ mice stereotactically injected with rotenone exhibited increased lesions and neuronal death, suggesting a neuroprotective role for the BVRA/bilirubin axis. Thus, bilirubin exerts both beneficial and deleterious effects, depending on its concentration.

## 3. Biliverdin Reductase-A in the Brain: More Than an Enzyme for Bilirubin Synthesis

In addition to its role in converting biliverdin to bilirubin, BVRA has additional protective roles in the brain [4,38]. Moreover, BVR expression is induced in neurons in response to ischemic injury. Specifically, BVR expression was increased in injured and neighboring neurons in a rodent model of stroke within six hours after middle cerebral artery occlusion (MCAO) [39]. This upregulation has been suggested to be related to the neuroprotective role of BVR in counteracting ROS after ischemia.

It has also been demonstrated that knocking down BVRA in rat primary neurons increases oxidative stress and elicits toxicity, further underscoring a role for BVRA in mitigating oxidative stress [14]. The subsequent generation of *Blvra*^−/−^ mice confirmed these findings, as these animals were found to be hypersensitive to oxidative stress, especially O_2_^·−^, leading to elevated lipid peroxidation and oxidative damage [8,34]. Thus, the depletion or inactivation of BVRA can have deleterious effects.

Indeed, in normal physiology, BVRA is highly versatile in its actions, participating in diverse physiologic processes [40,41]. In addition to its canonical role as the terminal enzyme in heme catabolism, BVRA also functions as both a kinase and a kinase substrate, such as for insulin receptor kinase [5,42,43,44,45], and additionally autophosphorylates itself. For example, BVRA phosphorylation is essential for its own reductase activity, which generates bilirubin. In addition, insulin receptor (IR) engagement phosphorylates BVRA at specific tyrosine residues, activating BVRA to function as a dual-specificity serine/threonine and tyrosine kinase that modulates several signaling events, including the signaling mediated by Akt and Erk [5,42]. BVRA also phosphorylates and thereby inhibits the insulin receptor substrate (IRS) to prevent the aberrant activation of IRS as part of a feedback regulatory loop. Furthermore, BVRA participates in a BVRA/glycogen synthase kinase 3β (GSK-3β)/peroxisome proliferator-activated receptor α (PPARα) axis that regulates hepatic lipid metabolism [46]. Here, GSK-3β phosphorylates its substrate, glycogen synthase 2 (GYS2), which is involved in glycogen production and storage in liver and muscle. GSK-3β phosphorylates GYS2 and inhibits its activity, leading to decreased glycogen storage [47]. Thus, glycogen storage is activated when GSK-3β is inhibited, which occurs when Ser 9 is phosphorylated by Akt [48,49]. GYS2 is also regulated at the transcriptional level by PPARα, a process inhibited by GSK-3β. GSK-3β phosphorylates Ser 73 of PPARα leading to its increased ubiquitination and protein turnover, as well as decreased activity [46]. Accordingly, liver-specific *Blvra*^−/−^ mice exhibited increased Ser 73 phosphorylation of PPARα, increased GSK-3β activity, and decreased glycogen storage, revealing a role for BVRA in inhibiting GSK-3β. Interestingly, bilirubin has been reported to inhibit many forms of protein phosphorylation that impact important cellular signaling processes [50,51,52,53]. How this might affect the above-mentioned feedback loop is an important area of future investigation. BVRA additionally modulates multiple metabolic and cellular pathways, including the control of glucose uptake, Akt signaling, immunosignaling, cellular proliferation, differentiation, and apoptosis [42,50,51,52,54]. Notably, signaling by BVRA has been shown to be disrupted in several diseases, as discussed below.

### 3.1. BVRA and Bilirubin in Alzheimer’s Disease

Alzheimer’s disease (AD) is the largest cause of dementia worldwide, with an estimated 413 million individuals on the AD continuum globally, of which 30 million have AD dementia [53]. AD may arise due to genetic causes or arise sporadically, with familial causes accounting for less than 5% of all cases [55].

The pathological hallmarks of AD include the deposition of intracellular neur ofibrillary tangles and paired helical fibrils composed of the protein tau and the accumulation of extracellular amyloid plaques composed of amyloid β (Aβ) peptides [56,57]. Tau is a microtubule binding protein that participates in axon outgrowth and neuronal transport [58,59]. Aberrant post-translational modifications on tau, such as hyperphosphorylation and acetylation, cause its dissociation from microtubules and aggregation, leading to neurotoxicity in AD and other forms of neurodegeneration [60,61,62,63]. The Aβ peptides are generated from the amyloid precursor protein (APP). APP can be cleaved at different sites by two major pathways: the nonamyloidogenic pathway and the amyloidogenic pathway. In AD, an increased production of the Aβ(1–42) fragment occurs by the action of beta-secretase 1 (BACE1) on APP [64,65,66]. AD has no cure, and existing treatments do not adequately treat disease symptoms. Recently approved therapies that target amyloid peptides and plaques have met with limited clinical success and are complicated by dangerous side effects to patients [67].

**Figure 3:**
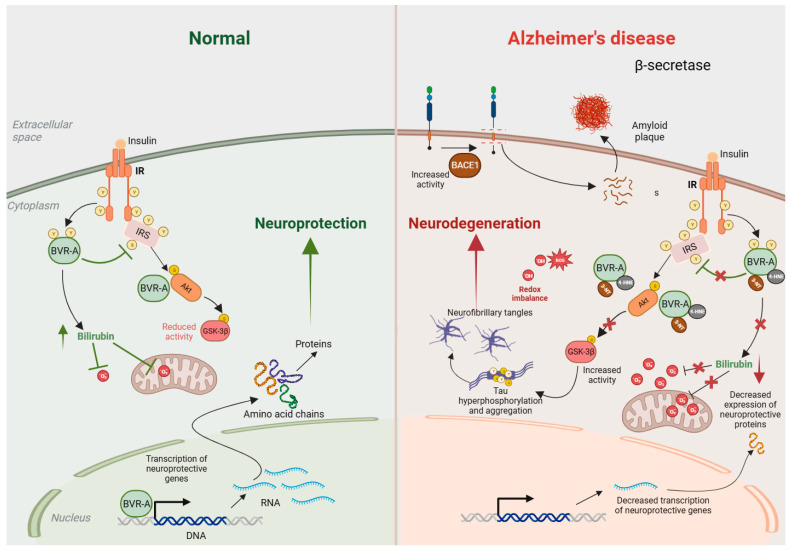
BVRA activity is decreased in Alzheimer’s disease (AD). Under normal conditions (**left**), insulin binds to the insulin receptor (IR) and stimulates auto-phosphorylation of IR on Tyr (Y) residues, which activates its kinase activity. Activated IR phosphorylates biliverdin reductase A (BVRA), as well as the insulin receptor substrate (IRS), on specific Y residues and activates them. Activated BVRA then phosphorylates and inhibits IRS itself, which prevents excessive IRS activation in a negative feedback loop. Activated IRS stimulates downstream signaling proteins, including phosphatidyl-inositol 3 kinase (PI3K), which activates Akt by phosphorylation. Activated Akt then phosphorylates GSK-3β on Ser9 and inhibits its kinase activity. BVRA additionally promotes Akt activation and GSK-3β inhibition by serving as a scaffold protein. Additionally, BVRA activity generates bilirubin, which scavenges superoxide (O_2_^·−^) and prevents oxidative and nitrosative damage. In AD (**right**), the activity of BVRA is low due to its inactivation by 4-hydroxynonenal (4-HNE) and nitration. This leads to an accumulation of reactive oxygen species (ROS) and reactive nitrogen species (RNS), which affect numerous cellular processes, including mitochondrial function. The loss of BVRA causes IRS hyperactivation and impaired insulin signaling. Downstream of IRS, suboptimal BVRA activity decreases Akt activation and GSK-3β inhibition, causing hyperphosphorylation of tau that leads to neurofibrillary tangle formation and neurotoxicity. Elevated ROS and RNS also favor tau and amyloid-β deposition. All these processes culminate in neurodegeneration.

One of the earliest studies that indicated the dysregulation of the BVRA/bilirubin axis in AD reported increased bilirubin levels in the cerebrospinal fluid (CSF) of patients [68]. Later, decreased plasma levels of antioxidants, including bilirubin, were identified as additional features of AD [69]. Moreover, the first enzyme in heme catabolism in the brain, heme oxygenase 2 (HO-2), is also dysregulated in AD. In addition, APP interacts with HO-2 and inhibits its activity, causing neurotoxicity [70]. Furthermore, cortical neuronal cultures from APP695swe transgenic mice have lower levels of bilirubin, which has been ascribed to lower HO-2 activity and lower biliverdin accumulation [70]. BVRA activity in AD was later analyzed by Barone and colleagues in 2011, who found decreased phosphorylation and increased oxidative/nitrosative post-translational modifications of BVRA in the hippocampus of patients with AD and mild cognitive impairment (MCI) [71,72,73]. These same modifications to BVRA were also observed in the plasma of AD patients and associated with increased plasma levels of BVRA and 3-nitrotyrosine-modification but decreased phosphotyrosine levels and BVRA reductase activity [74]. Furthermore, in a longitudinal study in the 3xTg-AD mouse model of AD, Barone and colleagues demonstrated that impaired BVRA activity promoted brain insulin resistance (BIR) and was one of the earliest pathologic events in this model [75]. A mechanism was proposed wherein oxidative-stress-mediated BVRA enzymatic impairment causes prolonged the activation of IRS, which triggers negative feedback mechanisms (such as those involving mTOR) that diminish autophagy and trigger IRS hyperactivity, thereby causing BIR [75,76,77]. The diminished activity of BVRA was also observed in other animal models of AD to lead to increased BACE1 phosphorylation, Aβ accumulation, and insulin resistance through downregulation of the insulin receptor (IR) and inhibition of IRS [77]. In line with these studies, the statin atorvastatin, which reduces the risk of AD, has also been shown to elevate the expression, phosphorylation, and activity of BVRA in the parietal cortex of aged beagles, a preclinical model of AD. Moreover, negative correlation between levels of BVRA, the accumulation of oxidative stress markers, and errors in discrimination learning were also observed in this model [78]. Notably, bilirubin treatment of a diet-induced model of obesity in mice reduced body weight and mitigated elevated blood glucose, total cholesterol (TC), leptin, and adiponectin levels, as well as normalized transcripts of sterol regulatory element-binding protein (SREBP-1), insulin receptor (IR), and PPARγ, and improved insulin sensitivity [79]. Independent studies also showed that either bilirubin levels or reductions in BVRA negatively correlated with obesity and its associated sequelae [80,81]. Lastly, signaling links between BVRA and neurodegeneration were established when it was shown that the loss of BVRA impaired the Akt-mediated inhibition of GSK-3β in response to oxidative stress, leading to tau hyperphosphorylation in early-stage AD [82]. The same study reported similar findings in MCI and also demonstrated that cells lacking BVRA exhibited lower GSK-3β inhibition and increased Tau Ser404 phosphorylation when subjected to oxidative stress. Thus, the BVRA/bilirubin axis is compromised in AD at multiple levels (Figure 3). An increased activation of GSK-3β has also been reported in the liver-specific *Blvra*^−/−^ mice, as described in Section 3 [46].

The aberrant expression and activity of BVRA have also been observed in several other conditions of impaired cognition. For example, BVRA levels were disrupted in the hippocampus, prefrontal cortex, and temporal lobe in a rat model of postoperative delayed neurocognitive recovery (dNCR) [83]. In addition to BVRA, the disposition of bilirubin is also compromised in several conditions, affecting brain function, as well as in various models of neurodegeneration, summarized in Table 1. Altered bilirubin levels have also been linked to neuropsychiatric disease. For instance, in male subjects with schizophrenia, total serum bilirubin levels were linked to cognition. Male patients with cognitive dysfunction had lower levels of total serum bilirubin [84]. An independent study reported an inverse correlation between lower bilirubin levels and working memory in first episode psychosis [85]. The same study also reported that lower bilirubin levels correlate with the duration of untreated psychosis (DUP). It must be noted, however, that while some studies reported an increase in either bilirubin levels or BVRA expression in brain diseases, others reported a decrease. For example, in a study involving drug-naïve PD patients, higher serum bilirubin levels were associated with better prognosis and outcomes over a two-year period [86]. Taken together, the date reflects perturbed bilirubin metabolism (Table 1).

### 3.2. BVRA and Synaptic Plasticity

**Figure 4:**
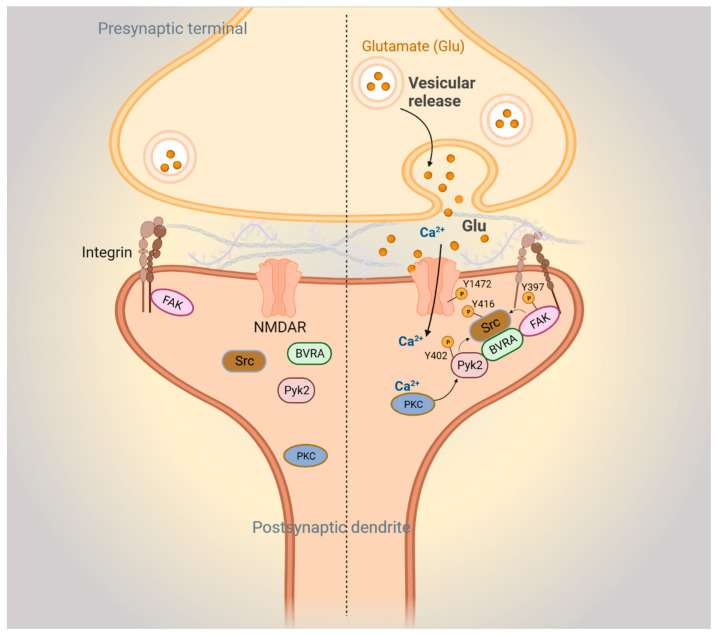
BVR-A mediates focal adhesion kinase-mediated synaptic signaling. Left: postsynaptic FAK-mediated signaling pathways during basal conditions. Right: FAK signaling during *N*-methyl-D-aspartate receptor (NMDAR)-mediated neurotransmission. Upon glutamate stimulation of the NMDAR, Ca^2+^ influx activates protein kinase C (PKC). Ca^2+^/PKC activates Pyk2 by stimulating autophosphorylation at Y402 and phosphorylation of integrins. The resulting conformational change in integrins activates autophosphorylation of FAK at Y397. Autophosphorylated Pyk2 and FAK then recruit and activate Src through phosphorylation at Y416, with BVRA acting as an adapter. Next, Src interacts with and phosphorylates the NR2B subunit of the NMDAR at Y1472, which further stimulates Ca^2+^ influx through the NMDAR.

Our laboratory has also shown that BVRA modulates synaptic function. First, we demonstrated that *Blvra*^−/−^ mice exhibited spatial learning deficits as assessed by the Morris water maze, which is dependent on intact hippocampal synaptic plasticity [98]. We also found that *Blvra*^−/−^ mice were compromised in fear conditioning as compared to wild-type (WT) littermates. Fear conditioning is a form of learning, distinct from spatial learning, in which anticipation of adverse events is learned. In a typical fear conditioning test, mice are trained to respond to a sound followed by a mild foot shock. With increasing tone–shock pair events, mice will increasingly freeze after the sound in anticipation of a foot-shock. The following day, the mice are returned to the same chamber. Mice that have associated the chamber with being shocked will freeze, whereas those that have not learned this association will not. Mice are then placed in a new chamber and exposed to the same sound from the previous day. Mice that have learned that the sound can precede a shock will freeze in fear, whereas those that were unable to learn this will not. In this test, young *Blvra*^−/−^ mice learned the cues and context as well as WT mice but had poorer recall memory, and aged *Blvra*^−/−^ mice were impaired in both learning and recall. Related to learning, a role for BVR has been established in synaptic plasticity. Synapses are dynamically modulated and can be structurally strengthened or weakened depending on the signal [99,100,101]. This plasticity has been linked to learning and memory involving various components of brain circuitry [102]. Under certain conditions, such as high-frequency stimulation, a sustained strengthening in synaptic transmission occurs, termed long-term potentiation (LTP), which regulates learning and memory [103]. An RNASeq analysis of the hippocampi of *Blvra*^−/−^ and WT mice revealed downregulation of focal adhesion kinase (FAK), which plays a central role in synaptic plasticity (Figure 4). FAKs are dynamic entities that link the intracellular cytoskeleton to the extracellular matrix [104]. FAK is a tyrosine kinase that signals alterations in the structure of focal adhesion complexes to key intracellular signaling hubs, such as phosphatidylinositol 3-kinase (PI3K) and mitogen-activated protein kinases (MAPKs), thereby transducing extracellular stimuli to intracellular signals that regulate synaptic strength [105,106,107,108]. BVRA facilitates this process by bridging focal adhesion signaling molecules at the synapse. BVRA also acts as an adaptor or bridge between the primary focal adhesion signaling kinases FAK and Pyk2 and the effector kinase Src (Figure 4). This activity is independent of BVRA’s catalytic activity. In the absence of BVRA, FAK and Pyk2 are unable to bind and stimulate Src, which is then unable to implement the phosphorylation of the NMDA receptor that is required for synaptic plasticity. As Src serves as a molecular hub where several signaling pathways converge to enhance NMDAR-mediated neurotransmission, BVR is positioned at a prominent intersection of synaptic signaling. Not surprisingly, the depletion of BVRA in hippocampal slices causes deficits in electrophysiological responses to stimuli.

## 4. Therapeutic Opportunities

As BVRA is unparalleled in its versatility of functions, it also offers opportunities for developing therapeutics at multiple levels. BVRA is positioned at the crossroads of a plethora of signaling cascades in the periphery and the brain and thus may provide a useful therapeutic target for a wide variety of diseases, including various forms of cancer, cardiovascular diseases, immune system disorders, and neurodegenerative diseases.

To date, BVRA-based peptide agonists have been considered for metabolic diseases. Specifically, a 7-residue peptide derived from the BVRA primary sequence can independently activate insulin receptor kinase autophosphorylation, kinase activity, and glucose uptake [50]. In addition to the periphery, BVRA also regulates insulin receptor signaling in the brain and prevents the hyperphosphorylation of tau through the Akt pathway [41,77,82]. Furthermore, BVRA levels are dynamically altered in response to insulin, including intranasal administration used to alleviate brain insulin resistance in AD [77,109]. In agreement with these reports, the loss of BVRA causes elevated oxidative stress, abnormal tau pathology, and impaired autophagy [8,14,34,76,82]. Autophagy impairment is a key feature of neurodegenerative diseases, and loss of BVRA impacts several aspects of autophagy. The reduced activity of AMP-activated protein kinase (AMPK), a key energy sensor that regulates cellular homeostasis and which is also an inhibitor of mTOR, is associated with loss of BVRA [76,110]. In line with these observations, it has been shown that high doses of biliverdin (the substrate of BVRA) activate the mTOR pathway, while rapamycin, an mTOR inhibitor, prevents this activation by biliverdin.

Several strategies may be adopted to augment BVRA levels and/or activity. For instance, peptides that activate the kinase and reductase activity of BVRA may be harnessed to mitigate neurodegeneration [111]. Earlier studies have revealed that BVR-derived peptides modulate functions related to kinase and scaffold activities of this enzyme. Three peptides derived from BVR have been characterized:^162^FGFPAFSG and ^275^KKRILHC and ^290^KYCCSRK. While the peptide KKRILHC inhibits BVRA kinase activity, the peptide KYCCSRK activates both the kinase and reductase activities [13,40,50,112]. In a preclinical model of cardiotoxicity, such as the perfused rat heart exposed to isoproterenol (ISO), the peptide KKRILHC (inhibitor) counteracted ISO-mediated BVRA activity and caused apoptosis and left ventricular dysfunction. On the other hand, perfusion with KYCCSRK (activator) augmented BVRA activity, prevented apoptosis, and preserved left ventricular function.

A significant challenge for delivering drugs to the brain involves the blood–brain barrier, and the development of lipid nanoparticles and cell-penetrating peptides derived from BVRA is an area that merits further investigation. As BVRA acts as an adaptor protein and participates in neurotransmission, augmenting the levels and activity of BVRA could optimize related processes as well [98]. In addition to these strategies, the use of bilirubin nanoparticles as therapeutics for metabolic and cardiovascular diseases has been considered [113,114,115]. In disorders with lowered bilirubin, suppressing the activity of UGT1A1 may provide an alternative approach to boost serum/plasma bilirubin levels [116]. In neurodegenerative disorders such as PD, in which bilirubin offers protection, inducers of BVRA and bilirubin protection are being considered. In a cell culture model of rotenone (an inhibitor of mitochondrial complex I) toxicity, in which bilirubin is depleted, BRUP-1-mediated modulation of bilirubin levels was found to be protective [117]. The use of such inducers of bilirubin production may be beneficial in other diseases such as AD in the future.

Screening for small molecules that upregulate the BVRA/bilirubin pathway could also result in the identification of novel therapeutic compounds. The administration of Atorvastatin (80 mg/day for 14.5 months) was reported to elevate BVR protein level, phosphorylation, and activity in the cortex of aged beagles, a preclinical model of AD [78]. The same study also reported that the induction of BVRA negatively correlated with the level of BACE1 protein and oxidative stress biomarkers, supporting a neuroprotective role for BVRA. Along similar lines with respect to the induction of BVRA protein, ferulic acid, a polyphenol abundant in fruits and vegetables, induces BVRA in theSH-SY5Y human neuroblastoma cell line and prevents oxidative stress-mediated neuronal death [118,119]. The augmentation of BVRA activity may also be beneficial in aging, the biggest risk factor for neurodegenerative diseases [120]. Centenarians, for example, are likely to display increased BVR-A levels in their blood [121]. Thus, stimulating the BVRA or augmenting bilirubin levels may be therapeutic in neurodegenerative diseases involving low bilirubin content.

In conclusion, the BVRA/bilirubin axis in the brain has, until recently, been an underappreciated system of natural neuroprotection that warrants further investigation and could serve to identify novel neuroprotective strategies for patients.

## Figures and Tables

**Table 1:** Summary of biliverdin reductase-A/bilirubin disposition in brain diseases.

Condition	Status of Bilirubin and BVRA	Reference
Alzheimer’s disease (3xTg-AD mouse model)	Decreased BVRA levels and activity, decreased Tyr-phosphorylation. Increased 3-NT modification of BVRA.	[75,87,88]
Alzheimer’s disease and MCI patients	Reduced BVR-A Tyr-phosphorylation and elevated 3-NT modification of BVR-A in the hippocampus.	[71,72,89]
Down Syndrome (Ts65dn mouse model)	Reduced BVR-A Tyr-phosphorylation in the frontal cortex (9 months).	[90]
Aging (C57BL/6J mice)	Decreased BVRA levels and phosphorylation (12 months) and increased 3-NT modification of BVRA (18 months) in the hippocampus.	[75]
Aging (Canine model)	Decreased BVRA Tyr-phosphorylation (4–12 months), elevated 3-NT modification of BVRA (10–12 months) in the parietal cortex.	[77]
Parkinson’s disease patients (without dementia)	Low bilirubin levels in serum of PD patients.	[91]
Parkinson’s disease (without dementia)	Higher bilirubin levels in plasma of PD patients.	[92]
Parkinson’s disease patients (de novo, drug-naïve)	Higher levels of bilirubin in serum, which correlated with better outcomes in a 2-year longitudinal study.	[86]
Amyotrophic lateral sclerosis (G93A SOD1 rat model)	Higher serum bilirubin levels in the symptomatic stage of ALS.	[93]
ALS patients	Lower serum bilirubin levels in patients with long duration ALS.	[94]
Migraine	Lower serum bilirubin in people with migraine.	[95]
Multiple sclerosis	Lower serum bilirubin in people with MS.	[96]
Multiple sclerosis (relapsing, remitting, or RRMS)	Lower serum bilirubin in people with MS	[97]

## Data Availability

Not applicable.
